# Regulation of MXene Membranes with β-Lactoglobulin Nanofiber-Templated CuS Nanoparticles for Photothermal Antibacterial Effect

**DOI:** 10.3390/polym17141960

**Published:** 2025-07-17

**Authors:** Zhuang Liu, Chenxi Du, Xin Zhou, Gang Wei

**Affiliations:** 1 School of Basic Medicine, Qingdao University, Qingdao 266071, China; zhuangliu001@outlook.com; 2 College of Chemistry and Chemical Engineering, Qingdao University, Qingdao 266071, China; duchenxi5@outlook.com; 3 School of Polymer Science and Engineering, Qingdao University of Science and Technology, Qingdao 266042, China

**Keywords:** MXene, hybrid membrane, CuS nanoparticles, photothermal effect, antibacterial materials

## Abstract

Developing advanced antimicrobial agents is critically imperative to address antibiotic-resistant infection crises. MXenes have emerged as a potential nanomedicine for antibacterial applications, but they suffer from suboptimal photothermal conversion efficiency and inherent cytotoxicity. Herein, we report the synthesis of MXene (Ti_3_C_2_)-based nanohybrids and hybrid membranes through firstly interfacial conjugation of self-assembled β-lactoglobulin nanofibers (β-LGNFs)-inspired copper sulfide nanoparticles (CuS NPs) onto MXene nanosheets, and subsequent vacuum filtration of the created β-LGNF-CuS/MXene nanohybrids. The constructed β-LGNF-CuS/MXene nanohybrids exhibit excellent photothermal conversion performances and satisfactory biocompatibility and minimal cytotoxicity toward mammalian cells, ascribing to the introduction of highly biocompatible β-LGNFs into the hybrid system. In addition, the fabricated β-LGNF-CuS/MXene hybrid membranes demonstrate high efficiency in antibacterial application through the synergistic photothermal and material-related antibacterial effects of both MXene and CuS NPs. Therefore, the ideas and findings shown in this study are useful for inspiring researchers to design and fabricate functional and biocompatible 2D material-based hybrid membranes for antimicrobial applications.

## 1. Introduction

Antimicrobial-resistant infections have become one of the most pressing public health concerns, primarily driven by the excessive use of antibiotics in clinical practice [1,2]. Bacteria frequently develop resistance through evolutionary adaptation during antibiotic treatment, thereby compromising the clinical utility of traditional antimicrobial therapies [3]. To address this challenge, researchers have been working on identifying and designing the next generation of highly active antimicrobial agents and biomaterials with low propensity for resistance development over the past few decades [4]. Advanced antimicrobial technologies currently being considered as alternatives to antibiotics include photodynamic therapy (PDT) using photosensitizers [5], chemodynamic therapy (CDT) using nanocatalysts [6,7], and photothermal therapy (PTT) using photothermal nanomaterials [8,9]. Two-dimensional (2D) transition metal carbides, carbonitrides, and nitrides (MXenes) are an emerging class of multifunctional nanomaterials that show unique advantages in PTT antimicrobial therapy [10].

MXenes exhibit an extraordinary photothermal conversion efficiency under near-infrared (NIR) laser excitation, enabling localized hyperthermia that induces simultaneous bacterial membrane disruption and protein denaturation [11]. This physical sterilization strategy rapidly destroyed pathogens and avoided the development of antimicrobial resistance [12,13]. However, MXene-based single PTT modalities showed inherent limitations, such as unsatisfying antimicrobial efficacy, and often fail to meet clinical needs [14,15]. Besides, the antibacterial mechanisms of MXenes may be less or even not effective against specific bacteria that have demonstrated high thermoresistance (e.g., *S. aureus*, which can tolerate temperatures as high as about 55 °C) [16]. Another limitation is the inevitable cytotoxicity of MXene, resulting from mechanical and oxidative stress, depending on the dose and duration of exposure [17]. To address these issues, Researchers have proposed synergistic strategies by combining MXene-mediated PTT with other therapeutic modalities or antimicrobial agents to reduce the dose of MXene [18,19]. For instance, MXene has been reported to act as both a photothermal agent and a photosensitizer carrier for synergistic PTT/PDT, with synergistic antibacterial activity against methicillin-resistant *S. aureus* [20]. This multimodal combination therapy can overcome the limitations of monotherapies and effectively suppress the resistance evolution of bacteria through synergistic interplay between distinct bactericidal modalities, providing a novel and promising approach for anti-infective therapy.

As a representative metal-based antimicrobial nanomaterial, copper (Cu)-based nanoparticles (NPs), such as Cu, CuO, Cu_2_O, and CuS, have attracted much attention for their broad-spectrum antimicrobial properties and have been applied in clinical scenarios such as wound dressings [21], orthopedic implant coatings [22], and antiviral fabrics [23]. Cu-based NPs exert bactericidal effects through dual synergistic mechanisms: (i) cationic surface interactions that compromise microbial membrane integrity via electrostatic disruption, and (ii) ROS-mediated oxidative cascade through the catalysis of hydrogen peroxide [24,25]. This coordinated action achieves potent microbicidal activity at low concentrations while circumventing conventional pathways of bacterial resistance. However, the cytotoxicity of Cu-based NPs remains an urgent issue, and the required dosage of Cu-based NPs for antibacterial applications is still higher than that of conventional antibiotics. In addition, Cu-based NPs exhibit a morphology-dependent photothermal activity and antibacterial efficiency, requiring a complicated synthesis procedure to enhance photothermal efficiency [26]. To enhance the antimicrobial performance, researchers have developed various functionalization strategies. For example, Cu-based NPs were conjugated with polymer biomaterials to construct hybrid systems or establish slow-release platforms through secondary carrier encapsulation [27,28]. Therefore, MXene-mediated combination therapy of PTT with Cu-based NPs holds great promise for antimicrobial applications. However, the clinical translation of Cu-based NPs still confronts two key barriers: (i) unselective interaction of Cu-based NPs with human cells causes off-target cytotoxicity [29], and (ii) the high surface energy promotes colloidal aggregation and thermodynamic instability [30,31], ultimately compromising antimicrobial efficiency and limiting the scope of clinical applications.

β-Lactoglobulin (β-LG) exhibits distinctive supramolecular assembly capabilities for optimizing metal-ion-based antimicrobial systems. Its sulfhydryl (-SH) and carboxyl (-COOH) groups enable strong coordination chelation of divalent metal ions, forming stable protein–metal supramolecular aggregates [32]. The protein shell of protein–metal nanocomposites blocks free Cu ion exposure and reduces the oxidative stress damage to mammalian cells, thereby enabling cytotoxicity regulation of Cu-based NPs [33]. Additionally, the amphiphilic structure of β-LG forms a dense stabilization layer on the surface of nanocomposites and generates a spatial site-blocking effect that effectively blocks the inter-particle van der Waals force interaction, thus contributing to the long-term stability of the colloidal dispersion system [34,35]. Compared with previously reported MXene–CuS and MXene–protein composites, our strategy employs β-lactoglobulin nanofibrils to facilitate spatially controlled CuS growth and hybridization with MXene, yielding membranes with enhanced PTT/CDT antibacterial efficacy and biocompatibility [36,37]. This interfacial engineering strategy based on protein conformation manipulation provides a new approach to enhance the biocompatibility and stability of Cu-based nanomaterials.

**Scheme 1:**
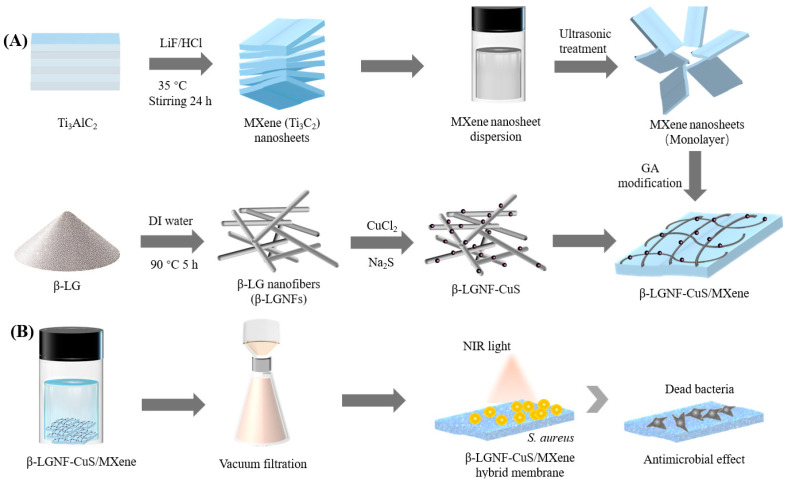
Schematics of preparations of (**A**) β-LGNF-CuS/MXene nanohybrids and (**B**) β-LGNF-CuS/MXene hybrid membranes for synergistic photothermal and material-related antibacterial application.

In this work, we fabricate antimicrobial MXene-based nanohybrids and hybrid membranes using vacuum filtration, which exhibit rapid bactericidal activity and sustained antibacterial efficacy through photothermal and material-based bacterial killing (Scheme 1). We first prepare β-LG nanofibers (β-LGNFs) by protein self-assembly to enhance the biocompatibility of the antimicrobial membrane and suppress CuS NP aggregation. Next, the pre-incubated β-LGNFs are used to synthesize CuS NPs by the adsorption of Cu^2+^ and the reaction with S^2−^, forming β-LGNF-CuS nanohybrids. As shown in Scheme 1A, glutaraldehyde (GA) is used as a cross-linking agent to conjugate MXene with β-LGNF-CuS to form β-LGNF-CuS/MXene nanohybrids, which are then vacuum filtered and dried to obtain a membrane-like structure (Scheme 1B). The coordinated antimicrobial effect of CuS NPs and MXene nanosheets endows the fabricated β-LGNF-CuS/MXene membranes with excellent antimicrobial activity against *S. aureus* and *S. epidermidis* via photothermal treatment while maintaining excellent biocompatibility due to the effect of β-LGNFs.

## 2. Materials and Methods

### 2.1. Materials

β-LG, copper chloride (CuCl_2_), sodium sulfide (Na_2_S), GA, dimethyl sulfoxide (DMSO), and L-ascorbic acid (AA) were purchased from Shanghai Macklin Biochemical Technology Co. (Shanghai, China). Ethanol, hydrochloric acid, hydrofluoric acid (HF), and sodium hydroxide (≥96%) were provided by Sinopharm Chemical Reagent Co. (Shanghai, China). Lithium fluoride (LiF), titanium aluminum carbide (Ti_3_AlC_2_), and sodium bicarbonate were purchased from Shanghai Yien Chemical Technology Co. (Shanghai, China).

### 2.2. Synthesis of Amyloid β-LGNFs and β-LGNF-CuS Nanohybrids

The synthesis of β-LGNFs was achieved by protein self-assembly. In brief, 0.04 g of β-LG powder was first dissolved in deionized (DI) water. Then, the solution pH was adjusted to 2.0 with 1 M HCl and diluted to 2% (*w*/*w*) using pH 2-adjusted DI water. The solution was then heated to 90 °C for 5 h with continuous stirring, at rates of 120 rpm for the first 3 h and 170 rpm for the last 2 h. The solution (β-LGNFs) was then cooled with ice and stored at 4 °C.

For the bioinspired synthesis of CuS NPs, CuCl_2_ (10 mM) was added to the β-LGNF solution in a 1:5 ratio (*v*/*v*) and stirred for 12 h. An equal volume of Na_2_S solution (10 mM) was then added to the stirred mixture to react with the Cu^2+^, resulting in the formation of β-LGNF-CuS nanohybrids.

### 2.3. Synthesis of MXene Nanosheets

MXene (Ti_3_C_2_) monolayered nanosheets were synthesized by HF etching of Ti_3_AlC_2_ and DMSO intercalation of multilayered MXene nanosheets based on a previous study [38]. Briefly, 1 mg of LiF was dissolved in 20 mL of HCl (12 M) and stirred for 10 min. 1 mg of Ti_3_AlC_2_ was then added to the mixture and stirred for 24 h at 35 °C. The solution was washed with 1 M HCl to remove unreacted LiF and impurities, followed by repeated washing with deionized water and centrifugation (8000 rpm, 5 min) until the pH of the solution reached 6.0–7.0. Monolayered MXene nanosheets were obtained by sonicating the suspension for 30 min. Subsequently, the supernatant was centrifuged, and the middle layer was collected for morphology characterization. Clay-like multilayer MXene, obtained through centrifugal precipitation, was added to DMSO and intercalated overnight under stirring. The solution was dialyzed with sodium ascorbate-containing dialysis buffer for 24 h to remove DMSO, and then freeze-dried to obtain monolayered MXene for further experiments.

### 2.4. Synthesis of β-LGNF-CuS/MXene Nanohybrids and Hybrid Membranes

A total of 10 mg of MXene powder was dispersed in 5 mL of sodium ascorbate solution. To further functionalize the MXene surface, 0.1% GA was added into the solution at a volume ratio of 1:10, followed by 12 h of stirring to yield GA-modified MXene. After centrifugation and thorough washing with deionized water (3 times) to remove free GA, 2 mg/mL GA-modified MXene dispersion was then mixed with an equal volume of β-LGNF-CuS nanohybrid solution. After 12 h of stirring, β-LGNF-CuS/MXene nanohybrids were created. The hybrid membranes were constructed through permeating β-LGNF-CuS/MXene nanohybrid solution on the surface of polyethersulfone (PES) membrane via vacuum filtration (0.08 MPa) for 12 h at room temperature.

### 2.5. Characterization Techniques

The prepared β-LGNFs and MXene nanosheets were characterized using atomic force microscopy (AFM) (FM-Nanoview 6800 AFM, FSM-Precision, Suzhou FSM Precision Instrument Co., Ltd., Suzhou, China) and transmission electron microscopy (TEM) (Tecnai G2 F20, FEI, Hillsboro, OR, USA). The microscopic structure of nanocomposites and membranes was observed via scanning electron microscope (SEM) (Regulus 8100, Hitachi, Tokyo, Japan). X-ray spectroscopy (XPS) (PHI 5000 VersaProbe III spectrometer, UlVAC-PHI, Chigasaki, Japan) was used to characterize the elemental composition of the samples.

To evaluate the structural durability of the β-LGNF-CuS/MXene hybrid membrane under physiologically relevant conditions, a cyclic stability test was performed. The membrane was immersed in 0.9% NaCl solution and subjected to near-infrared (NIR) laser irradiation (808 nm, 1.0 W/cm^2^) for 5 min per cycle, once per day, over a total period of 7 days. After each cycle, the membrane was gently rinsed with deionized water and air-dried at room temperature before the next cycle [39]. Mechanical properties of the hybrid membranes after thermal cycling were assessed via uniaxial tensile testing using a universal testing machine (AGS-X, SHIMADZU, Shanghai, China) at a strain rate of 5 mm/min.

### 2.6. Controlled Release of Cu^2+^

Cu^2+^ ion release was quantified by ICP-MS. Hybrid membranes (1 cm^2^) were immersed in 5 mL of 0.9% NaCl solution at 37 °C, with or without NIR irradiation (808 nm, 1.0 W/cm^2^, 5 min per cycle). At predetermined time points (1, 3, 5, and 7 days), aliquots of the supernatant were collected and filtered through a 0.22 μm membrane before analysis using inductively coupled plasma-mass spectrometry (ICP-MS, Agilent 7900, Tokyo, Japan).

### 2.7. In Vitro Biocompatibility of β-LGNF-CuS/MXene Nanohybrids

MC3T3 cells were cultured in α-MEM medium supplemented with 10% fetal bovine serum. To assess the biocompatibility of the materials, 2 × 10^5^ cells were seeded in 24-well plates and incubated for 12 h. β-LGNF-CuS/MXene nanohybrids were added to the cell medium at a concentration of 10 μg/mL. Cells were incubated for an additional 24 h and 72 h and then stained using Calcein AM/PI Assay Kit (Beyotime, Shanghai, China) according to the manufacturer’s protocol.

### 2.8. In Vitro Antibacterial Assay

*S. aureus* and *S. epidermidis* were cultured in Luria–Bertani (LB) agar plates at 37 °C. Control, MXene, and hybrid membranes were added into the medium and cocultured with bacterial suspension overnight to detect the inhibitory effect of the materials on bacterial growth. For the PTT group, a mixture of samples and *S. aureus* or *S. epidermidis* was exposed to NIR irradiation (808 nm, 1.5 W/cm^2^) for 30 min. The colonies were counted to quantify the antimicrobial effect of the materials.

## 3. Results and Discussions

### 3.1. Synthesis and Characterizations of β-LGNFs

**Figure 1:**
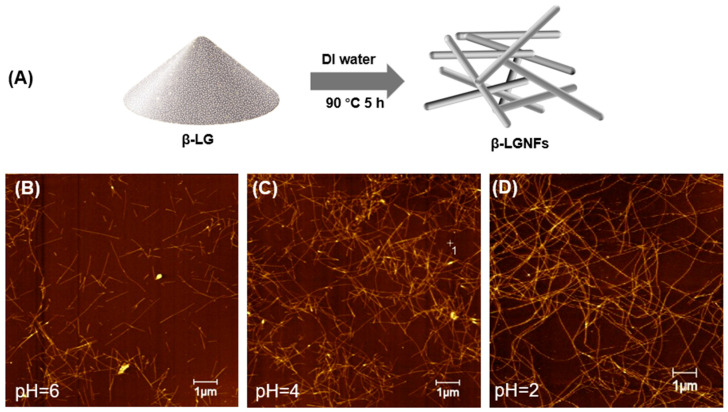
Synthesis and AFM characterization of β-LGNFs. (**A**) Schematic synthesis. (**B**–**D**) AFM height images of β-LGNFs incubated at different pH values: (**B**) pH 6, (**C**) pH 4, and (**D**) pH 2.

With its abundant functional amino acid residues, amyloid β-LGNFs serve as both reducing agents—capable of directly reducing alloy nanoparticles—and as protective agents that prevent NP aggregation and enhance the dispersive stability of CuS NPs. These fibers also impart excellent biocompatibility and multifunctionality to the composite system, serving as an efficient carrier for antimicrobial materials. Thermal treatment under low pH and low ionic strength conditions is the most commonly employed method to induce fibrogenesis in β-LG, significantly accelerating the rate of protein self-assembly (Figure 1A) [40,41].

We then compared the morphological features of β-LGNFs that were synthesized under varying pH conditions (Figure 1B–D). The proteins exhibited markedly different aggregate morphologies depending on pH. As the pH decreased, the β-LGNFs underwent distinct morphological transformations. The fiber diameter increased, the fibers elongated, and the degree of entanglement and aggregation intensified, resulting in a denser and more complex network structure. These transformations are likely associated with pH-induced conformational changes in the protein and modifications in intermolecular interactions. At pH 2, β-LGNFs showed a high degree of aggregation, with notable thickening of fiber diameter, elongation, and intertwining, ultimately forming a stable network structure. These results suggest that enhanced intermolecular forces under these highly acidic conditions induced extensive protein molecular aggregation and structural reorganization of the protein nanofibers.

### 3.2. Synthesis and Characterizations of β-LGNF-CuS Nanohybrids

**Figure 2:**
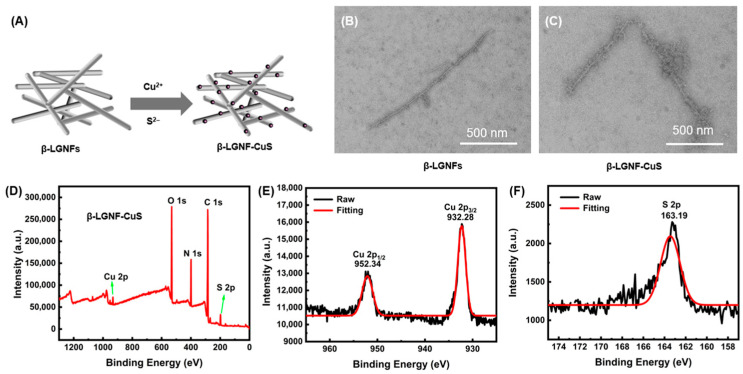
Synthesis and characterization of nanofibers and β-LGNF-CuS nanohybrids. (**A**) Schematic synthesis process. (**B**,**C**) TEM images of β-LGNFs and β-LGNF-CuS nanohybrids. (**D**–**F**) XPS spectrum of (**D**) β-LGNF-CuS, as well as spectra of (**E**) Cu 2P and (**F**) S 2p.

Subsequently, β-LGNFs prepared by incubation at pH 2 were utilized as templates for the synthesis of β-LGNF-CuS nanohybrids via a bioinspired synthesis method (Figure 2A) [42]. Their morphological characteristics were subsequently analyzed using TEM. The created β-LGNF exhibited elongated and morphologically uniform fiber structures with relatively smooth surfaces (Figure 2B). This homogeneous morphology and surface smoothness may enhance the mechanical properties and dispersibility of the fibers in practical applications. In β-LGNF-CuS hybrids, most CuS NPs were uniformly distributed along the surface of the β-LGNFs, although minor agglomeration was observed (Figure 2C). The deposition of CuS NPs increased the surface roughness of the nanofibers, resulting in an increased specific surface area and consequently offering more active sites for catalytic processes.

To further confirm the formation of CuS NPs on the synthesized β-LGNFs, XPS characterization was performed. The XPS spectrum of the β-LGNF-CuS nanohybrids (Figure 2D) revealed that the nanohybrids contain signals corresponding to Cu 2p, O 1s, N 1s, C 1s, and S 2p. In particular, the detailed XPS spectra of Cu 2p (Cu 2p_1/2_ at 952.34 eV and Cu 2p_3/2_ at 932.28 eV) and S 2p (at 163.19 eV) further confirmed the successful nucleation and growth of CuS NPs onto the β-LGNF (Figure 2E,F) [43,44]. Based on these results, it can be concluded that β-LGNF-CuS nanohybrids were successfully synthesized.

### 3.3. Synthesis and Characterization of MXene Membranes

**Figure 3:**
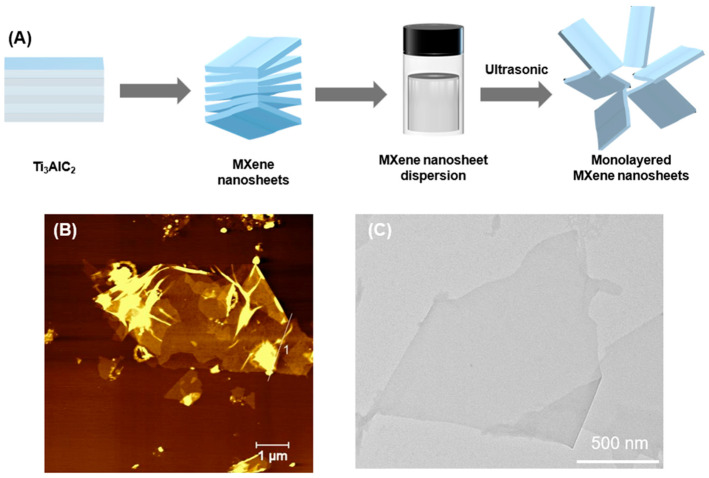
Synthesis and characterization of MXene nanosheets. (**A**) Schematic illustration of MXene synthesis. (**B**,**C**) AFM and TEM images of MXene nanosheets.

Two-dimensional materials have great application potential in areas including electronic devices, energy storage, and biomedical devices [45,46]. Two-dimensional monolayered MXene nanosheets were synthesized via HF etching (Figure 3A) [47]. The prepared MXene materials were characterized using AFM and TEM to evaluate their morphology and size in detail. AFM image revealed that the MXene nanosheets exhibit a highly planar surface morphology (Figure 3B). TEM analysis further confirmed the presence of a transparent, film-like single-layer MXene structure with a flat surface and well-defined edges (Figure 3C). These results demonstrate that the MXene monolayer nanosheets prepared by this method possess excellent 2D structural characteristics, with their high transparency and sharp edges indicating high structural integrity and minimal defect density.

**Figure 4:**
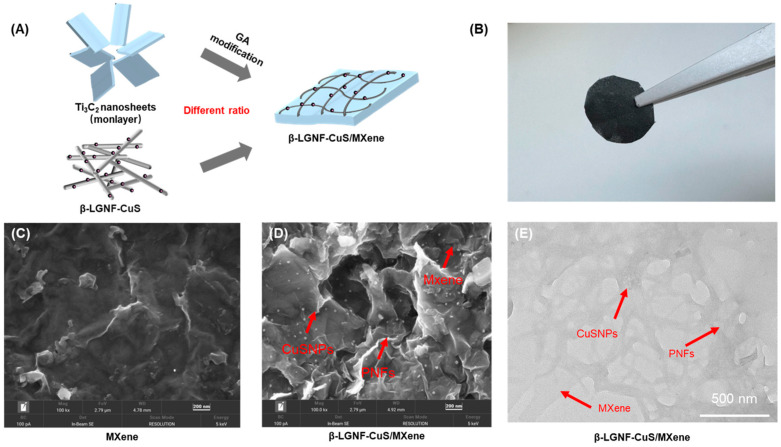
Synthesis and structural characterization of β-LGNF-CuS/MXene hybrid membranes. (**A**) Conjugation of β-LGNF-CuS and GA-MXene. (**B**) Digital photograph of β-LGNF-CuS/MXene membrane. (**C**) SEM image of MXene nanosheets. (**D**) SEM and (**E**) TEM images of β-LGNF-CuS/MXene hybrid membranes.

β-LGNF-CuS/MXene hybrid membranes were synthesized as illustrated in Figure 4A. After the fabrication of β-LGNF-CuS/MXene hybrid membrane, a photographic image of the resulting membrane shows a uniform black appearance (Figure 4B). The microstructure of the material was further analyzed using SEM and TEM. The MXene nanosheets exhibited a typical layered architecture with a relatively smooth surface and well-defined edges, which is characteristic of 2D materials (Figure 4C). Compared to MXene, SEM and TEM images of the β-LGNF-CuS/MXene hybrid membranes (Figure 4D,E) reveal a uniform distribution of CuS NPs across the MXene nanosheet surface, resulting in the formation of a stable composite structure. Additionally, β-LGNFs promoted the mechanical stability of the final membrane through the interactions with both CuS NPs and MXene nanosheets, we suggest.

Considering the importance of membrane stability in practical applications, the stability of the β-LGNF-CuS/MXene composite membrane was investigated under cyclic near-infrared (NIR) irradiation and in physiological saline (Figure S1, Supporting Information). After 7 days of cyclic NIR exposure and immersion in physiological saline, the hybrid membrane maintained its structural integrity without any visible deformation or delamination, indicating excellent resistance to photothermal stress and ionic environments [39]. Uniaxial tensile tests showed that the tensile strength of the composite membranes after photothermal cycling was 473.6 kPa. (Figure S2, Supporting Information).

### 3.4. Photothermal Properties and Cu^2+^ Release Profile of β-LGNF-CuS/MXene Nanohybrids

**Figure 5:**
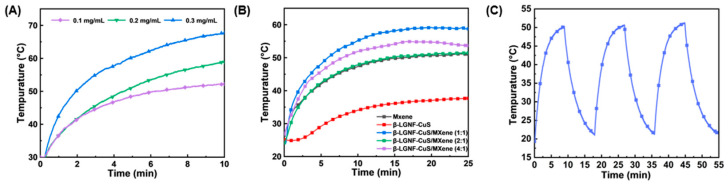
Photothermal performance of β-LGNF-CuS/MXene nanohybrids under NIR irradiation (808 nm, 1.5 W/cm^2^). (**A**) Temperature elevation of β-LGNF-CuS/MXene at varying concentrations (1.5 W/cm^2^). (**B**) Heating and cooling curves of various samples. (**C**) Photothermal stability of β-LGNF-CuS/MXene nanohybrids (0.2 mg/mL) over three heating-cooling cycles.

To evaluate the photothermal properties of β-LGNF-CuS/MXene nanohybrids, a laser with a wavelength of 808 nm and a power density of 1.5 W/cm^2^ was used to irradiate suspensions of varying concentrations and compositional ratios. The results demonstrated that the temperature increase of the nanohybrids increased with the suspension concentration, confirming the excellent photothermal conversion capability of the β-LGNF-CuS/MXene hybrids (Figure 5A). Specifically, at a concentration of 0.2 mg/mL, the suspension temperature increased to 58.9 °C within 10 min under NIR light irradiation. Notably, the incorporation of β-LGNF-CuS nanohybrids did not significantly alter the intrinsic photothermal performance of MXene (Figure 5B). Furthermore, the photothermal effect of the β-LGNF-CuS/MXene hybrids remained stable after three consecutive heating-cooling cycles, indicating its excellent photothermal stability (Figure 5C). These favorable photothermal characteristics suggest that the β-LGNF-CuS/MXene hybrids show high potential as efficient PTT reagents, with promising applications in localized thermotherapy, particularly in enhancing antimicrobial efficacy. Additionally, the results from ICP-MS indicated that exposure under NIR significantly promoted the release of Cu^2+^ from the β-LGNF-CuS/MXene membrane (Figure S3, Supporting Information).

### 3.5. Biocompatibility of β-LGNF-CuS/MXene Nanohybrids

**Figure 6:**
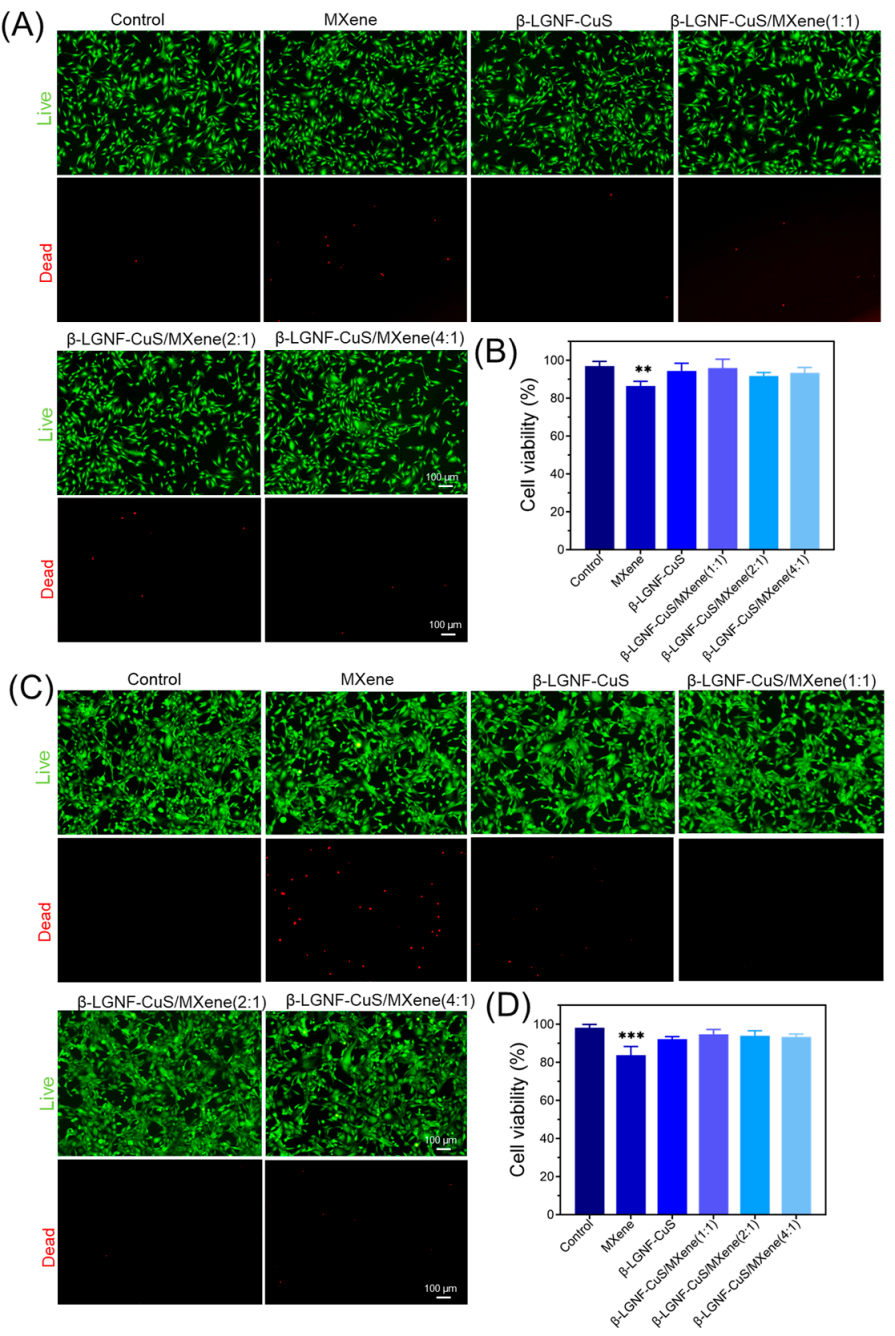
In vitro biocompatibility assay of β-LGNFs-CuS/MXene nanohybrids. (**A**) Fluorescent photographs of live/dead cell staining after 24 h. (**B**) The quantification of cell viability after 24 h. (**C**) Fluorescent photographs of live/dead cell staining after 72 h. (**D**) The quantification of cell viability after 72 h. (*n* = 3) The data are presented as mean ± standard deviation (SD). ** *p* < 0.01, *** *p* < 0.001.

Favorable biocompatibility is imperative for medical devices and clinical translation [48]. Cell live/dead staining was performed to investigate the cytotoxicity of the β-LGNFs-CuS/MXene nanohybrids. As shown in Figure 6A, most materials had no significant effect on cell proliferation after 24 h. However, partial cell death was observed in the MXene group, with a cell survival rate of approximately 86.50% (*p* < 0.01). The toxic effects of MXene may be attributed to apoptosis due to cellular internalization and cell membrane rupture due to MXene-cell contact [49]. Despite minor cell death in some fields of view, most cells retained good morphology and viability, with survival > 90% in the β-LGNFs-CuS and β-LGNFs-CuS/MXene group (Figure 6B). After 72 h, the cell viability in the MXene group decreased to 83.82%, while most of the cells in the β-LGNFs-CuS and β-LGNFs-CuS/MXene groups were still alive. (Figure 6C,D). These results suggest that the protein shell of β-LGNFs-CuS greatly enhances the biocompatibility of CuS NPs. Besides, the interfacial conjugation of β-LGNFs-CuS overcomes the high cytotoxicity of conventional MXene materials. Therefore, we demonstrate that β-LGNFs-CuS/MXene nanohybrids have good biocompatibility, which provides inspiration for future MXene-based membrane materials in biomedical applications.

### 3.6. Antibacterial Properties of β-LGNF-CuS/MXene Hybrid Membranes

**Figure 7:**
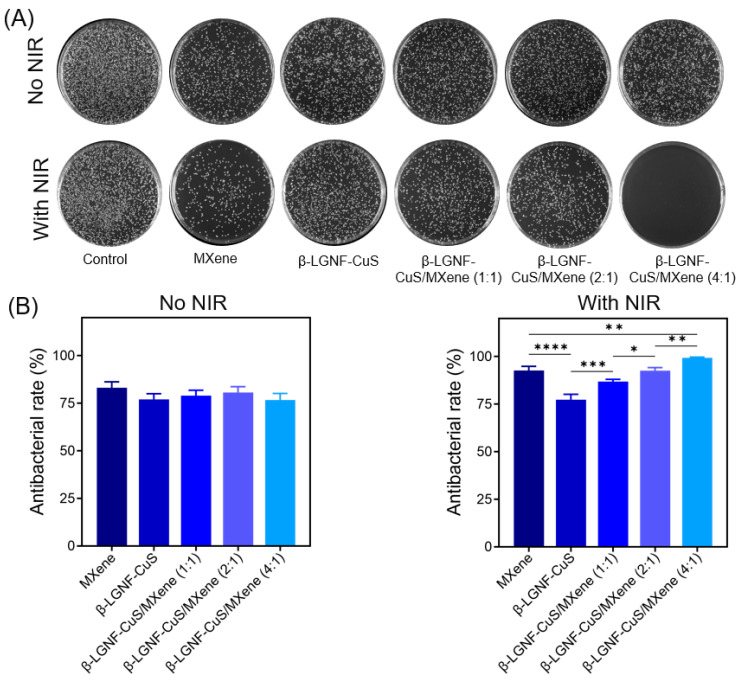
Antibacterial assay of hybrid membranes. (**A**) Agar plate photographs of *S. aureus*. (**B**) The quantification of the antibacterial rate. (*n* = 3) The data are presented as mean ± standard deviation (SD). * *p* < 0.05, ** *p* < 0.01, *** *p* < 0.001, **** *p* < 0.0001.

Finally, *S. aureus* was chosen to verify the antibacterial ability of the β-LGNFs-CuS/MXene hybrid membranes. As shown in Figure 7A, antibacterial efficacy was observed in all samples. As previously reported, the sharp outer edges of MXene disrupt bacterial biofilms, leading to bacterial rupture and inactivation [50]. The physical disruption and Cu^2+^ release are the main factors for bacterial killing. However, in the absence of NIR light irradiation, there was no significant difference in the antimicrobial rate (about 75–80%) between all the materials (Figure 7A). In the PTT group (with NIR light irradiation), the antimicrobial properties of all materials except β-LGNF-CuS were enhanced. This is consistent with the poor photothermal performance of β-LGNF-CuS in the above-presented photothermal results. It should be noted that in the presence of NIR light irradiation, the sample of β-LGNF-CuS/MXene (4:1) showed the greatest antibacterial property (nearly 99%) (Figure 6B). While higher β-LGNF-CuS loading reduced photothermal conversion, we demonstrate that the ratio of 4:1 could be the optimal ratio to amplify the synergistic effect of β-LGNFs and MXene for a satisfactory antimicrobial effect. The results support that the synergistic effect of the physical disruption, Cu^2+^ release, and photothermal effect achieves the greatest antimicrobial efficacy. In addition, to demonstrate the efficacy of clearance against other significant pathogens, we also investigated the antimicrobial properties of β-LGNF-CuS/MXene (4:1) against *S. epidermidis* (Figure S4, Supporting Information). β-LGNF-CuS/MXene (4:1) exhibited > 95% antimicrobial efficiency against *S. epidermidis* under NIR light irradiation. Despite the promising antibacterial performance and biocompatibility of β-LGNF-CuS/MXene nanohybrids, potential challenges remain in scaling up this technology for clinical translation. These include the need for precise control of CuS nucleation during large-scale synthesis, and ensuring long-term storage stability. Furthermore, regulatory hurdles for clinical approval of nanomaterials must also be addressed before practical application.

## 4. Conclusions

In summary, we reported the bioinspired, template-assisted synthesis of β-LGNFs-CuS nanohybrids, which were subsequently conjugated with MXene nanosheets to form β-LGNFs-CuS/MXene nanohybrids. Hybrid membranes were then fabricated using vacuum filtration. The resulting β-LGNFs-CuS/MXene nanohybrid exhibited high structural integrity and uniform distribution. As a result, the synthesized nanohybrids presented high photothermal conversion efficiency and excellent biocompatibility, and the fabricated hybrid membranes revealed effective antibacterial activity against *S. aureus* and *S. epidermidis*. Therefore, the proposed organic–inorganic hybrid membranes represent a promising strategy for the design and development of MXene-based antibacterial biomaterials with improved performance. Future research should focus on further optimizing the structure–property relationships of β-LGNFs-CuS/MXene hybrid membranes, especially under in vivo conditions. It is also important to investigate long-term antibacterial efficacy, biodegradation behavior, and immune responses in relevant animal models. In addition, exploring scalable fabrication methods and expanding the hybrid system to other types of pathogenic bacteria could broaden its biomedical applicability.

## Data Availability

Data are contained within the article and Supplementary Materials.

## Supplementary Materials

The following supporting information can be downloaded at https://www.mdpi.com/article/10.3390/polym17141960/s1, Figure S1: β-LGNF-CuS/MXene composite membrane after cyclic near-infrared (NIR) irradiation and physiological saline immersion. Figure S2: Mechanical properties of hybrid membranes after thermal cycling. Figure S3: Release curve of Cu^2+^ in β-LGNF-CuS/MXene composite membrane. Figure S4: Antibacterial effect of hybrid membranes for *S. epidermidis*. (A) Agar plate photographs of *S. epidermidis*. (B) The quantification of the antibacterial rate. (*n* = 3) The data are presented as mean ± standard deviation (SD).
