# Supplementary material for: Regulation of MXene Membranes with β-Lactoglobulin Nanofiber-Templated CuS Nanoparticles for Photothermal Antibacterial Effect
Source: Polymers (Basel). 2025 Jul 17;17(14):1960. doi: 10.3390/polym17141960 (PMC12300359; doi:10.3390/polym17141960)
Supplement: Supplementary file 1 [file polymers-17-01960-s001.zip › polymers-3733754-supplementary.pdf]

Supplementary Materials

Regulation of MXene Membranes with β-Lactoglobulin Nanofiber-Templated CuS Nanoparticles for Photothermal Antibacterial Effect

Zhuang Liu [1,2], Chenxi Du [2,3], Xin Zhou [1,2,*] and Gang Wei [3*]

[1] School of Basic Medicine, Qingdao University, 266071 Qingdao, China; zhuangliu001@outlook.com (Z.L.)
[2] College of Chemistry and Chemical Engineering, Qingdao University, 266071 Qingdao, China; DuChenxi5@outlook.com (C.D.)
[3] School of Polymer Science and Engineering, Qingdao University of Science and Technology, 266042 Qingdao, China
* Correspondence: wei-lab@qust.edu.cn (G.W.), wei@uni-bremen.de (G.W.), Tel.: +86-15066242101; zhouxin@qdu.edu.cn (X.Z.)

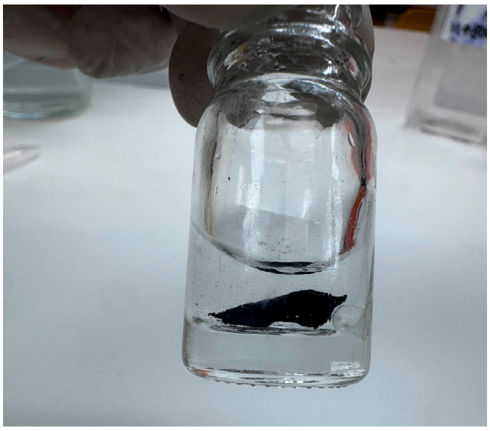

Figure S1. β-LGNF-CuS/MXene composite membrane after cyclic near-infrared (NIR) irradiation and physiological saline immersion.

Supplementary Materials

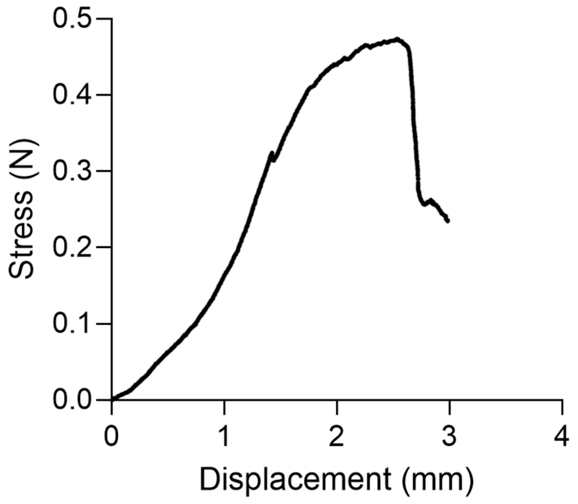

Figure S2. Mechanical properties of hybrid membranes after thermal cycling.

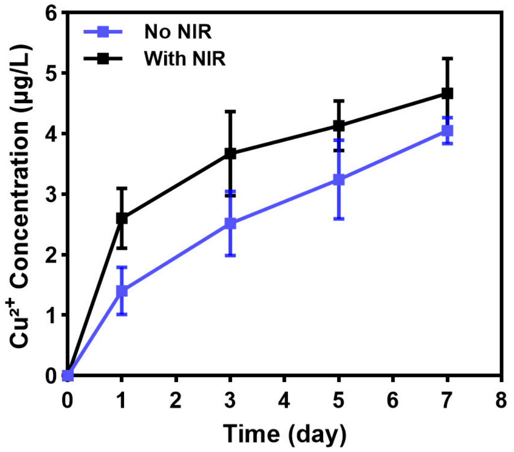

Figure S3. Release curve of Cu^{2+} in β-LGNF-CuS/MXene composite membrane.

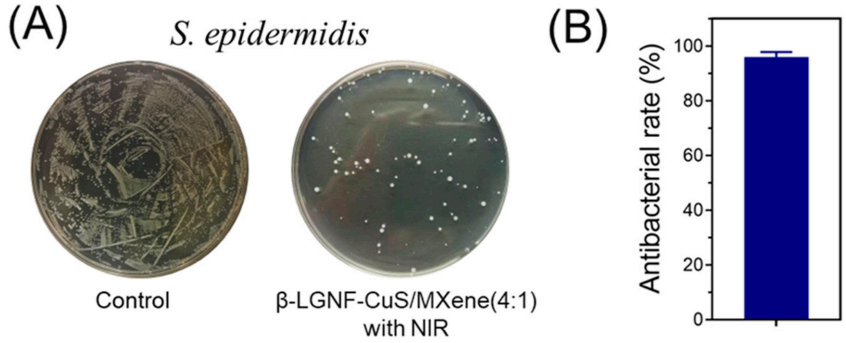

Figure S4. Antibacterial effect of hybrid membranes for *S. epidermidis*. (A) Agar plate photographs of S. epidermidis. (B) The quantification of the antibacterial rate. (n=3) The data are presented as mean ± standard deviation (SD).
